# Ocean Wave Simulation Based on Wind Field

**DOI:** 10.1371/journal.pone.0147123

**Published:** 2016-01-25

**Authors:** Zhongyi Li, Hao Wang

**Affiliations:** School of Hydropower and Information Engineering, Huazhong University of Science and Technology, Wuhan, Hubei Province, China; CNRS, FRANCE

## Abstract

Ocean wave simulation has a wide range of applications in movies, video games and training systems. Wind force is the main energy resource for generating ocean waves, which are the result of the interaction between wind and the ocean surface. While numerous methods to handle simulating oceans and other fluid phenomena have undergone rapid development during the past years in the field of computer graphic, few of them consider to construct ocean surface height field from the perspective of wind force driving ocean waves. We introduce wind force to the construction of the ocean surface height field through applying wind field data and wind-driven wave particles. Continual and realistic ocean waves result from the overlap of wind-driven wave particles, and a strategy was proposed to control these discrete wave particles and simulate an endless ocean surface. The results showed that the new method is capable of obtaining a realistic ocean scene under the influence of wind fields at real time rates.

## Introduction

Ocean simulation techniques are widely applied in the fields of virtual reality, 3D video games and so forth. Describing ocean waves requires a high degree of complexity, not only because the wavelengths of ocean waves range from millimeters to kilometers but also because ocean wave movement is related to wind, tide and terrain. While today’s ocean simulation techniques are highly realistic, presenting a simple and efficient approach to simulate realistic ocean scenes in real time remains a challenge.

A large number of contributions and improvements on ocean simulation have been achieved. In early work, some authors used the method of summing a series of periodic functions to represent an ocean surface [1–4]. While this method offers lower fidelity than subsequent methods of ocean simulation, it remains a choice for some programmers due to the merits of fast and stable computation, high controllability and inexpensive computation cost.

Rather than deterministic entities, other researchers treat ocean waves as statistical processes. For instance, Mastin et al. [5] applied the Pierson-Moskowitz spectrum [6], and Premoze and Ashikhmin [7] used the JONSWAP spectrum [8]. Following these authors, Tesserdorf [9] used the spectral components obtained from the Phillips spectrum [10] and a Gaussian pseudo-random generator to achieve a realistic result, and such approaches have become a classical method of ocean simulation. Mitchell [11] implemented Tesserdorf’s method on GPU by employing a low-frequency band to displace the geometry of the ocean surface and a broad band to represent fine ocean details with interactive frame rates. LeBlanc et al. [12] further divided the broad wave spectra into several narrow bands to decrease the computational cost with superior visual results.

In addition, particle system techniques are a popular method for simulating fluids, and Reeves [13] first introduced such techniques to simulate clouds, water, fire and smoke. Peachey [2] used particle systems to represent spray, which is produced by breaking waves and collisions with obstacles. The Smoothed Particle Hydrodynamics (SPH) model [14] has been used in various applications to simulate fluids: animating the soft bodies of lava flow [15], rendering the free surface of low compressibility fluids [16] and representing the splashing behavior of viscoelastic fluids, such as paint and mud [17]. Highly realistic simulation results have been produced by these physically based methods at the cost of complex computation, which leads to limited use in simulating large bodies of water.

Our method resembles the particle system method introduced by Yuksel et al. [18] in that the wave particles are also applied into height field construction. However, the authors in [18] mainly focused on the interaction of ocean waves and solid objects instead of on ocean waves themselves. The method we proposed is a fresh attempt to apply the particle system techniques. The particles, called wave particles in our paper, are driven by field data, an artificial data which is called wind field. Through introduce the wind field data to the constructing process of the ocean height, as well as to management and controlling of wave particles, all types of ocean waves under the influence of wind fields can be obtained by our method.

## Methods

For an observer, the ocean surface can be treated as the result of the superposition of wind waves with various properties, including position, propagation direction, speed and shape. We hope to directly relate wind force to the formation and propagation processes of ocean waves, a mechanism that is extremely complex in the real world. However, it should be clear that the the formation and propagation processes of ocean waves driven by wind force we mentioned is the simplication of the similar processes in physical environment. It means that the physical mechanism that wind force driving ocean waves is out of consideration in this paper, and physical-accurate model to simulate endless ocean surface in real time is impossible for current graphic hardware. The main aim of our paper is to construct a simple, efficient and useful ocean scene which can be applied in computer graphic, rather than a physical model that simulates ocean surface height field. Therefore, the main challenges of our method are how to represent ocean waves and how to reflect wind in the behaviors of ocean waves. It is our objective to solve these difficulties in obtaining realistic ocean scenes.

We begin our explanation of our method with the mathematical description of ocean waves, introducing wave particles to represent wind waves, in the first subsection. The second subsection describes how to construct a realistic height field for the ocean surface under the influence of a wind field.

### Definition of wave particle

The ocean surface can be represented by a dynamic height field *Y*(*x*, *z*, *t*), which is the deviation amplitude at time *t* in the horizontal position *X* = (*x*, *z*). Ocean waves, which are generated by wind force, propagate with velocity *v* and satisfy a second order wave equation:

$$\frac{\partial^{2}Y}{\partial x^{2}}+\frac{\partial^{2}Y}{\partial z^{2}}=\frac{1}{v^{2}}\frac{\partial^{2}Y}{\partial t^{2}}$$ (1)

In our method, we utilize and expand the idea of particle systems to represent ocean waves as wave particles. Wave particles contain all relevant properties of ocean waves. Applying wave particles makes it convenient to receive wind information so that we can easily manage and control ocean waves.

The shape of an individual wave particle is represented by the deviation function *D*_*i*_(*x*, *z*, *t*). Hence, the dynamic height field of ocean can be described as

$$Y\left(x,z,t\right)=\sum_{i}D_{i}\left(x,z,t\right)$$ (2)

$$D_{i}\left(x,z,t\right)=a_{i}\left(t\right)\times W_{i}\left(x,z,t\right)$$ (3)

where *W*_*i*_(*x*, *z*, *t*) is the waveform function, the amplitude of the *i*^*th*^ particle *a*_*i*_(*t*) is given by

$$a_{i}\left(t\right)=\sin\left(\pi \times \frac{t-t_{born}}{t_{span}}\right)\times amp_{i}$$ (4)

where *t*_*born*_ is the time when particles are generated, *t*_*span*_ is the time span from birth time to dead time of particle, and *amp*_*i*_ is the maximum amplitude. Eq 4 reflects the formation, development and dying process of a wave particle. As each ocean wave moves, the amplitude increases to the maximum amplitude *amp*_*i*_ and then decreases to zero at time *t*_*dead*_. A stronger wind force causes the wave particle to move with higher maximum amplitude and energy, so *amp*_*i*_ can be formulated as

$$amp_{i}=k_{a}\times \left|V_{i}\right|$$ (5)

where **V**_**i**_ is the wind propagation direction. The choice of *k*_*a*_ is arbitrary: any positive number is acceptable.

Either a mathematical model or a statistical model assumes that the ocean surface can be considered as a combination or superposition of a large number of regular sinusoidal wave components with different properties. According to this assumption, the sinusoidal wave is the natural choice of the waveform function. In addition to providing a similar shape to the ocean wave, the waveform function *W*_*i*_(*x*, *z*, *t*) should also fulfill other requirements as follows:

- The deviation values of the waveform function must be zero at boundary points to ensure that the waveform function is continuous at boundary areas.
- Considering the lighting computation when we need to render a wave particle, the first derivative of the deviation values of the waveform function versus position should be continuous.

Thus, we choose

$$W_{i}\left(x,z,t\right)=\frac{1}{2}\times \cos\left(\pi \times \frac{dist}{r_{i}}+1\right)\prod \left(\frac{dist}{2r_{i}}\right)$$ (6)

$$\prod \left(\frac{dist}{2r_{i}}\right)=\left\{\begin{array}{c} 1,\left|\frac{dist}{2r_{i}}\right|<\frac{1}{2}\\ \frac{1}{2},\left|\frac{dist}{2r_{i}}\right|=\frac{1}{2}\\ 0,\text{otherwise} \end{array}\right.$$ (7)

as our waveform function, where *r*_*i*_ is the radius of wave particle, $\prod (\frac{dist}{2r_{i}})$ is a rectangle function. *dist* = |(*x*, *z*) − (*x*_*i*_(*t*), *z*_*i*_(*t*))| is the distance between the sampled point (*x*, *y*) and the central point of particle (*x*_*i*_(*t*), *z*_*i*_(*t*)). The position of a wave particle is represented by the position of the central point of the wave particle, and the change in particle location is influenced by the wind propagation direction, which can be described by

$$\left(x_{i}\left(t\right),z_{i}\left(t\right)\right)=\left(x_{i}\left(0\right),z_{i}\left(0\right)\right)+D_{i}\times C_{i}\times t$$ (8)

where (*x*_*i*_(0), *z*_*i*_(0) is the initial position of the *i*^*th*^ wave particle, **D**_**i**_ is the propagation direction of wave particle which perpendicular to the wave front along which the crest travels, and *C*_*i*_ is the propagation velocity of wave particle to represent the distance the crest moves forward per second.

Both of propagation velocity and direction of wave particle are closely related to the the wind direction **V**_**i**_. Although we will be occupied mostly by a ocean wave simulation method based on wind field and wave particles, we begin by introducing a method presented by Tessendorf [9] to describe how the motions of wave particles are affected by wind. Let us to describe the ocean surface using a spectral distribution of waves, obtained from theoretical or measured data. The wave height *h*(**x**, *t*) at the horizontal position **x** = (*x*, *z*) as the sum of sinusoids with complex, time-dependent amplitudes:

$$h\left(x,t\right)=\sum_{k_{i}}\tilde{h}\left(k_{i},t\right)exp\left(ik_{i}\cdot x\right),$$ (9)

where *t* is the time and **k**_**i**_ is a two-dimensional vector, called the wavevector, which is a horizontal vector that points in the direction of travel of the wave. The magnitude of wavevector, wavenumber *k*_*i*_, which is inversely proportional to the wave length λ_*i*_ as

$$\lambda_{i}=2\pi /k_{i}.$$ (10)

Statistical analysis of a number of wave-buoy, photographic, and radar measurements of the ocean surface demonstrates that the wave height amplitudes $\tilde{h}\left(k_{i},t\right)$ are nearly statistically stationary, independent, gaussian fluctuations with a spatial spectrum denoted by

$$P_{h}\left(k_{i}\right)=\langle |{\tilde{h}}_{0}^{*}\left(k_{i},t\right){|}^{2}\rangle ,$$ (11)

for data-estimated ensemble averages denoted by the brackets 〈〉. For wind-driven waves in a fully developed sea, the spectrum *P*_*h*_(**k**_**i**_) is given by

$$P_{h}\left(k_{i}\right)=A\frac{exp(-1/{\left(k_{i}\lambda_{i}\right)}^{2})}{{k_{i}}^{4}}{\left|k_{i}\cdot W\right|}^{2},$$ (12)

where **W** is the normalized direction of the wind, *A* is a numeric constant.

Eq 12 provides us two important points. Firstly, the cosine factor |**k**_**i**_ ⋅ **W**|^2^ in the Phillips spectrum eliminates waves that move perpendicular to the wind direction. The mathematical description of Phillips spectrum reflects that, the smaller the angle between the wind direction and the propagation direction of ocean wave, the larger the value *P*_*h*_(**k**_**i**_). The cosine factor |**k**_**i**_ ⋅ **W**|^2^ in the spreading function ensures that waves travel predominantly in the direction of the wind. Therefore, it is reasonable that the wind direction can be treated as the propagation direction of wave particles to represent the ocean waves which are the most significant in ocean surface, as described as Eq 13

$$D_{i}=W=normalize\left(V_{i}\right)$$ (13)

Secondly, λ_*i*_ = |**V**_**i**_|^2^/*g* is the largest waves that carry the most energy arising from a continuous wind of speed |**V**_**i**_|. Such formulation indicates the relationship between wind speed and wavelength of ocean wave, thus can further relate wind speed to the propagation velocity of wave particle. Specially, according to Eq 10, the wavenumber *k*_*i*_ can be calculated as

$$k_{i}=\frac{2\pi }{\lambda_{i}}=\frac{2\pi g}{|V_{i}{|}^{2}}.$$ (14)

Additionally, in the deep, open ocean, the wave dispersion relation

$$\omega \left(k_{i}\right)=\omega_{i}=\sqrt{gk_{i}},$$ (15)

relates the angular wave frequency to the wave number. Meanwhile, the angular frequency *ω*_*i*_ is also related to the period *T*_*i*_ by

$$\omega_{i}=\frac{2\pi }{T_{i}}.$$ (16)

On the other hand, in the implementation procedure of our method, the wave particles are emitted from the *particle origin* at a constant time interval Δ*t*_*i*_ (the relevant contents are specially presented in the next section), and the wave crests are located in the central points of wave particles. Thus, the distance, which is actually the wavelength of ocean waves λ_*i*_, between wave particles can be described as

$$\lambda_{i}=\Delta t_{i}\times C_{i}.$$ (17)

The well-known dispersion relationship between wavelength and wave propagate speed is written as

$$\lambda_{i}=T_{i}\times C_{i}.$$ (18)

The period of ocean waves *T*_*i*_, therefore, can be calculated as Δ*t*_*i*_. Thus, according to Eqs 14, 15 and 16, the period can be formulated as

$$T_{i}=\Delta t_{i}=\sqrt{2\pi }\times \frac{|V_{i}|}{g}.$$ (19)

As a result, the relationship between the propagation velocity of wave particle and the wind speed is given by

$$C_{i}=\lambda_{i}/T_{i}=\frac{{|V_{i}|}^{2}}{g\times T_{i}}=\frac{1}{\sqrt{2\pi }}\times \left|V_{i}\right|.$$ (20)

**Fig 1:**
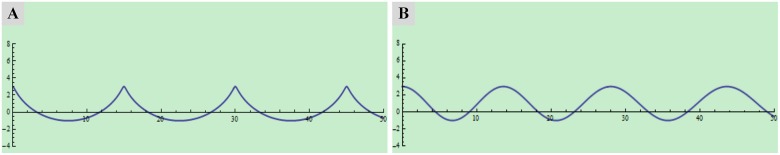
Comparison between two-dimensional Gerstner waves and sinusoidal wave. (A) Gerstner waves with longitudinal component. (B) Sinusoidal wave without longitudinal component.

**Fig 2:**
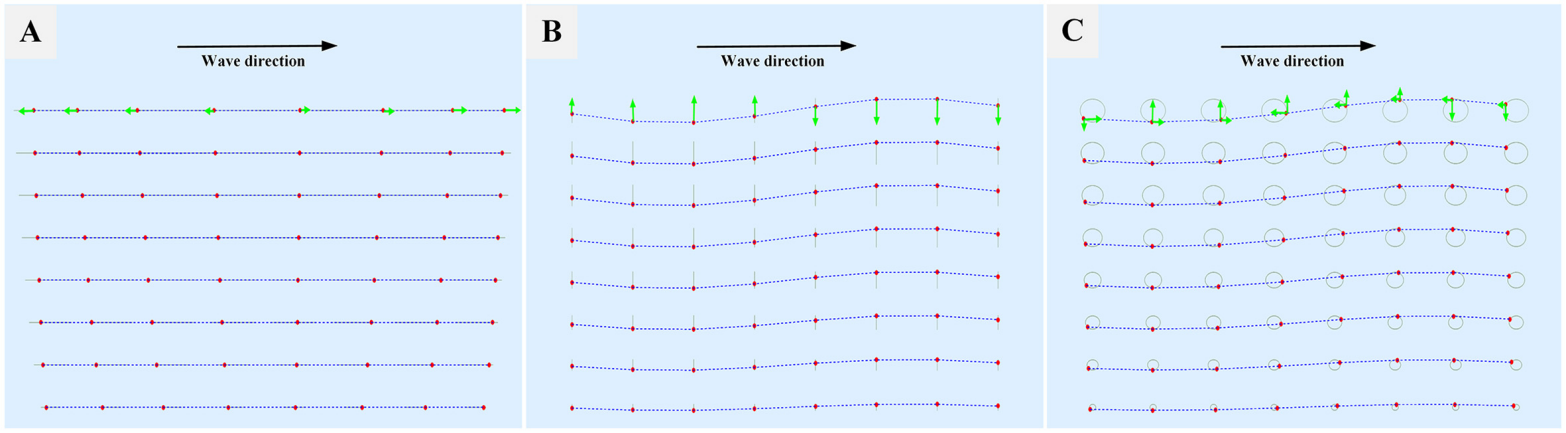
Longitudinal and transverse components of ocean waves. (A)The longitudinal wave. (B) The transverse wave. (C) The ocean wave with longitudinal and transverse components. The red dots indicate the momentary position of fluid particles on their (gray) orbits. The green arrows indicate the directions of motions of particles, and the blue dashed line are isopressure lines. The radius of this circular motion, in (C), is the largest on the ocean surface and this radius exponentially decreases deeper into the water volume.

Nonetheless, the ocean surface cannot be represented solely by the sum of sine waves in vertical direction. The shape of an ocean wave is often depicted as a sine wave with vertical motion only, but the actual shape should be described as a trochoidal shape with both vertical and horizontal motions. Fig 1 shows the comparison and difference between the Gerstner wave and the sinusoidal wave. The Gerstner waves model [19] is a physical model to describe the motion of particles on ocean surface. The particles move in circles when a wave passes by. Specially, the motion of water surface is forward as the peak of the ocean wave passes, but backward as the trough of the wave passes, arriving again at the same position when the next peak arrives. Compared with the crest of the Gerstner wave, the crest of the sinusoidal wave is less sharper, and the sinusoidal wave trough is less smooth than the through of the Gerstner wave. The difference of wave shapes between the Gerstner wave and the sinusoidal wave is due to the fact that the Gerstner wave is composed of both transverse and longitudinal waves. Fig 2 illustrates the transverse and longitudinal components of ocean waves, as well as how the transverse component is aligned with the longitudinal. There is a constant phase relationship between the transverse and the longitudinal components. With respect to the direction of the oscillation relative to the propagation direction, we can distinguish between longitudinal wave and transverse wave.

Hence, the lateral movement of vertices in the extended height field can be described by

$$H{}_{xz}\left(x,z,t\right)=\sum_{i}\;D{}_{i}^{L}\;\left(x,z,t\right)$$ (21)

where $D{}_{i}^{L}\left(x,z,t\right)$ is the longitudinal deviation on the ocean surface, which can be formulated as

$$D{}_{i}^{L}\left(x,z,t\right)=a_{i}\left(t\right)\times W{}_{i}^{L}\;\left(x,z,t\right)$$ (22)

The state of each longitudinal wave as a function of horizontal position (*x*, *z*) and time *t* is defined as

$$W{}_{i}^{L}\;\left(x,z,t\right)=\left\{\begin{array}{c} -\sin\left(\frac{\pi \times \left(x-x_{i}\left(t\right)\right)}{r_{i}}\right)\times W_{i}\left(x,z,t\right)\\ -\sin\left(\frac{\pi \times \left(z-z_{i}\left(t\right)\right)}{r_{i}}\right)\times W_{i}\left(x,z,t\right) \end{array}\right.$$ (23)

Therefore, for any (x, y) in the 2D horizontal plane, the 3D position P on the ocean surface is:

$$P\left(x,z,t\right)=\left\{\begin{array}{c} x+\sum_{i}D{}_{i}^{L}\;\left(x,z,t\right).x\\ \sum_{i}D_{i}\left(x,z,t\right)+D_{noise}\left(x,z,t\right)\\ z+\sum_{i}D{}_{i}^{L}\;\left(x,z,t\right).z \end{array}\right.$$ (24)

### Construction of wind-driven ocean waves

**Fig 3:**
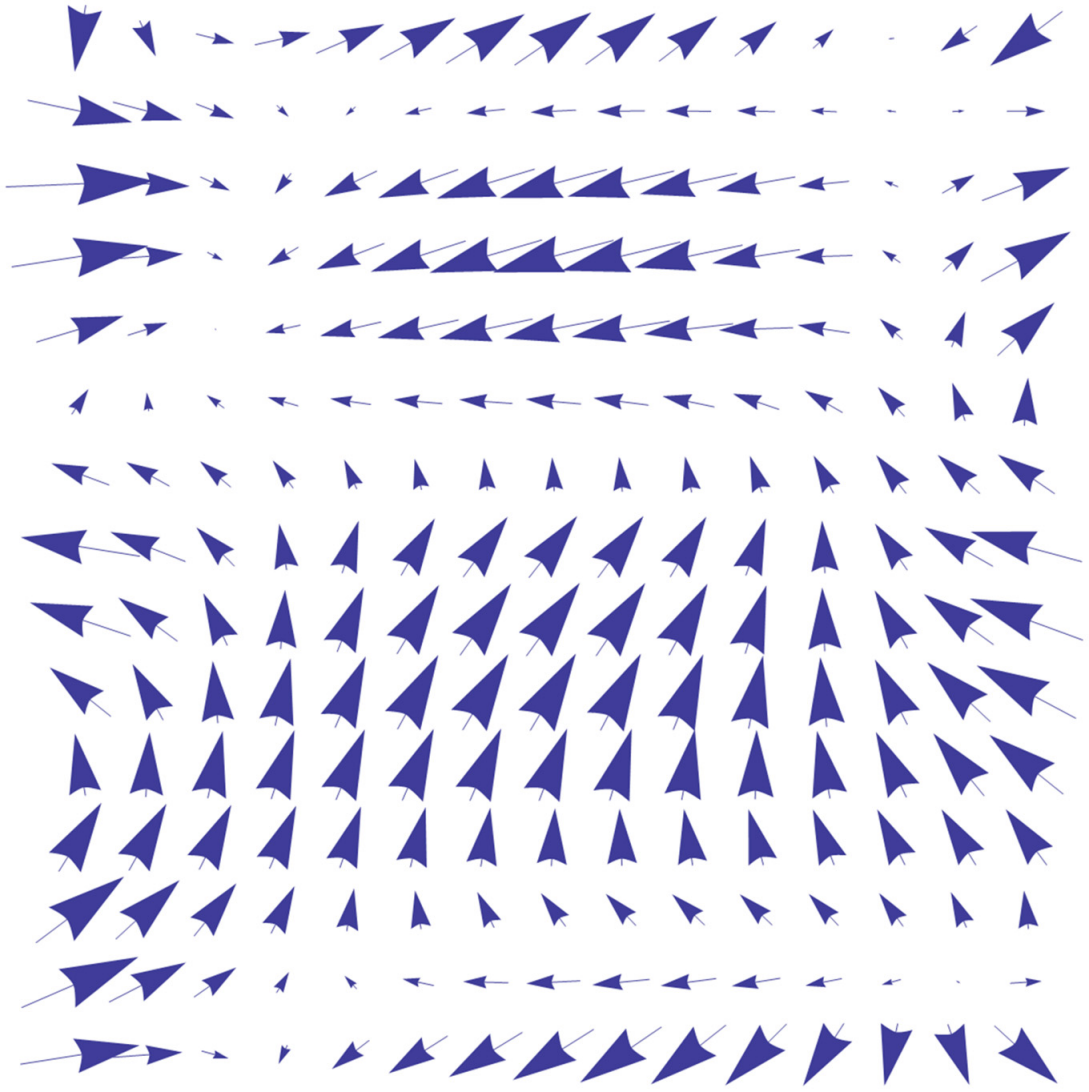
Wind field data. The data structure of the wind field is vector data. The strength of the wind force can be described by the size of the arrow of the wind field, in addition to the information on the direction of propagation.

The wind force is the main energy resource to generate ocean waves, and waves cannot exist by themselves. We begin our explanation of the process that transforms wind energy to ocean surface movement with two concepts: *wind field* and *particle origin*. The *wind field*, in oceanography and meteorology, is actually a vector field that can be used to describe wind information, such as direction and velocity, at any discretely sampled position (see Fig 3). But in our method, the *wind field* is actually an artificial vector field. This means that through setting different wind field data, the ocean surface height field influenced by such wind field can be simulated. The *particle origin* does not actually exist in the ocean scene, but is a medium for wind force to drive ocean waves in our method. The process by which wind force drive ocean waves, therefore, can be described as follows: based on the wind data from the wind field, the particle origins initialize and generate wave particles which are constantly updated according to their properties assigned by particle origins and which represent the wind waves in the ocean surface.

#### Distribution of particle origins

Because the wave particles independently update their positions and phases of life, the chaotic movement of wave particles without global control strategies would affect the realism of the ocean scene.

Because the initialized positions of wave particles are directly related to the positions of their particle origins, the distribution of particle origins affect the distribution of wave particles on the ocean surface. Therefore, we divide a certain area of ocean equally into *N* × *M* small patches (*N* rows and *M* columns), with one or a few particle origins in each patch. This procedure causes the distribution to disperse wave particles uniformly across the ocean surface. Without this restriction on the distribution, the wave particles would most likely gather in some areas, leading to excessive amplitudes of ocean waves. Meanwhile, there might be fewer or even no wave particles in some other areas.

#### Emitting of ocean waves

The particle origins emit the ocean waves, which each consist of several wave particles instead of a single one. The dispersive wave particles cannot form the integral and continuous ocean wave that should result from the effective overlap between particles.

**Fig 4:**
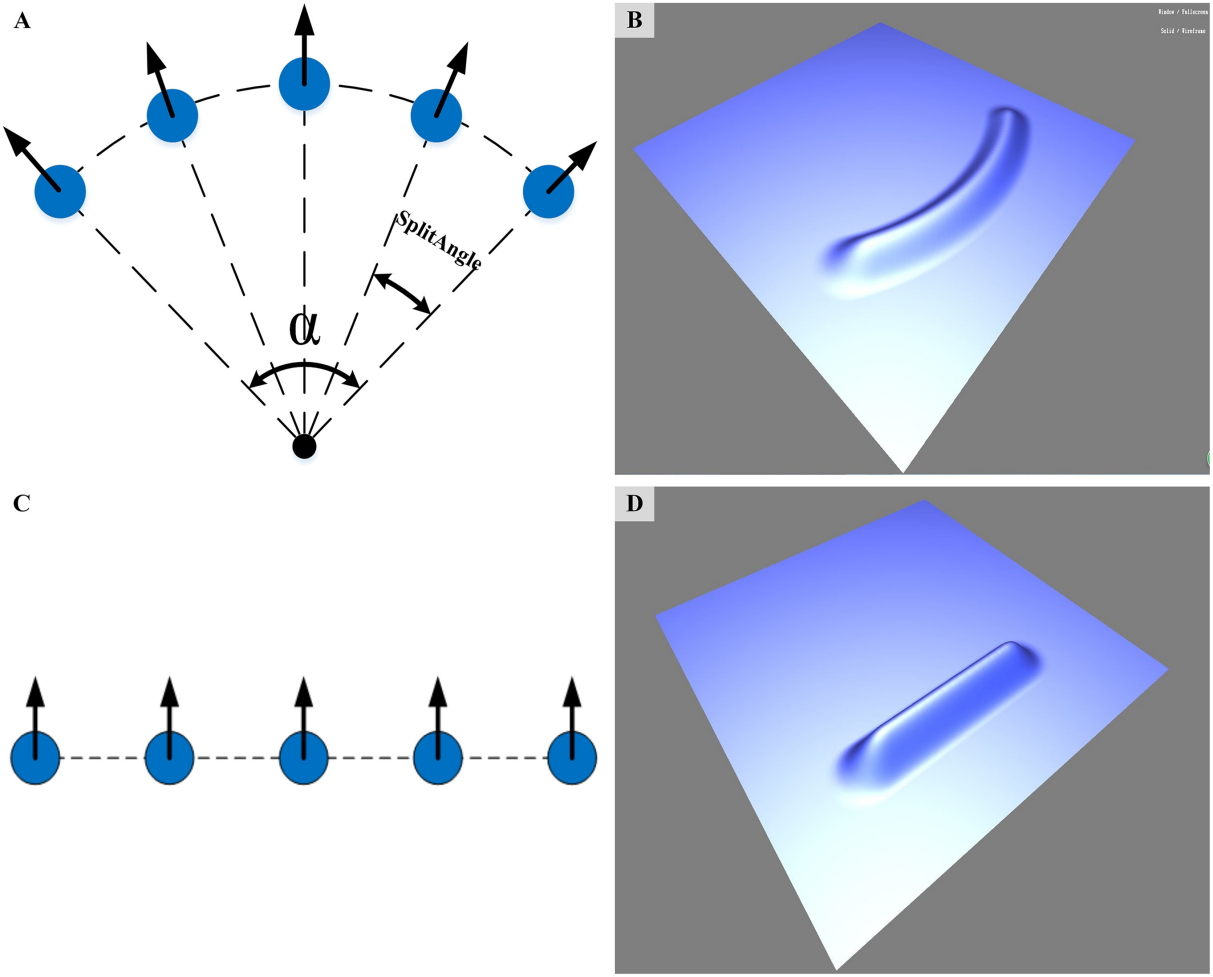
Initial wavefront shapes emitted from particle origin. (A) and (B) are the position of the wave particles and the radial wavefront shape they represent. (C) and (D) are the position of the wave particles and the parallel wavefront shape they represent.

Concerning the initial wavefront shape emitted from the particle origin, Fig 4 shows us two options. Fig 4A shows that wave particles emitted from the same particle origins at the same time are exactly on the same circular arc, and the middle particle gains the propagation direction from its particle origin. *SplitAngle* is the offset between the propagation directions of neighboring particles, so the radial wavefront (Fig 4B) still moves along the direction assigned by the wave origin. The wave particles in Fig 4C are placed side by side with identical propagation velocity and direction, and the relevant wavefront is shown in Fig 4D.

We tend to choose the wavefront shape in Fig 4B because the angle *α* can change the shape of the curved wavefront, and the curved shape is similar to the result of interaction between water surface and wind, whereas the wavefront illustrated in Fig 4D looks artificial. Note that *α* should be small, as a larger angle produces an excessively curved wavefront shape. Additionally, if *α* is large, the distance between neighboring particles would increase rapidly and thus split more unnecessary new particles. The particle splitting procedure is discussed in the next subsection.

#### Splitting of wave particles

**Fig 5:**
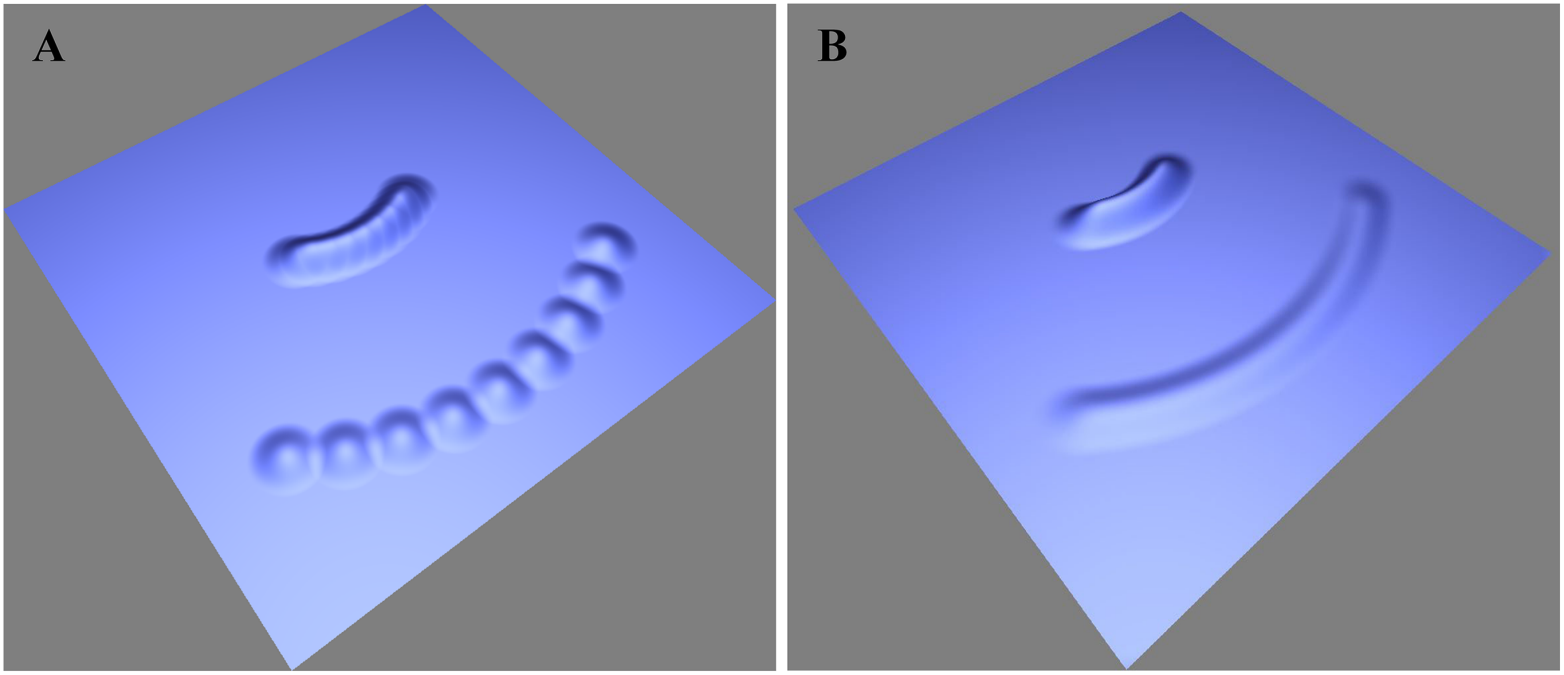
Comparison between wavefronts with and without splitting of particles. (A) The wavefront without splitting after the wave particles travel some distance. (B) The wavefront with splitting after the wave particles travel some distance.

The radial wavefront presents a problem when the wave particles travel some distance after they are emitted. Because the propagation directions of neighboring wave particles are different, the distance between neighboring particles will increase gradually, and the continuous wavefront will split into several dispersive particles, as shown in Fig 5A.

**Fig 6:**
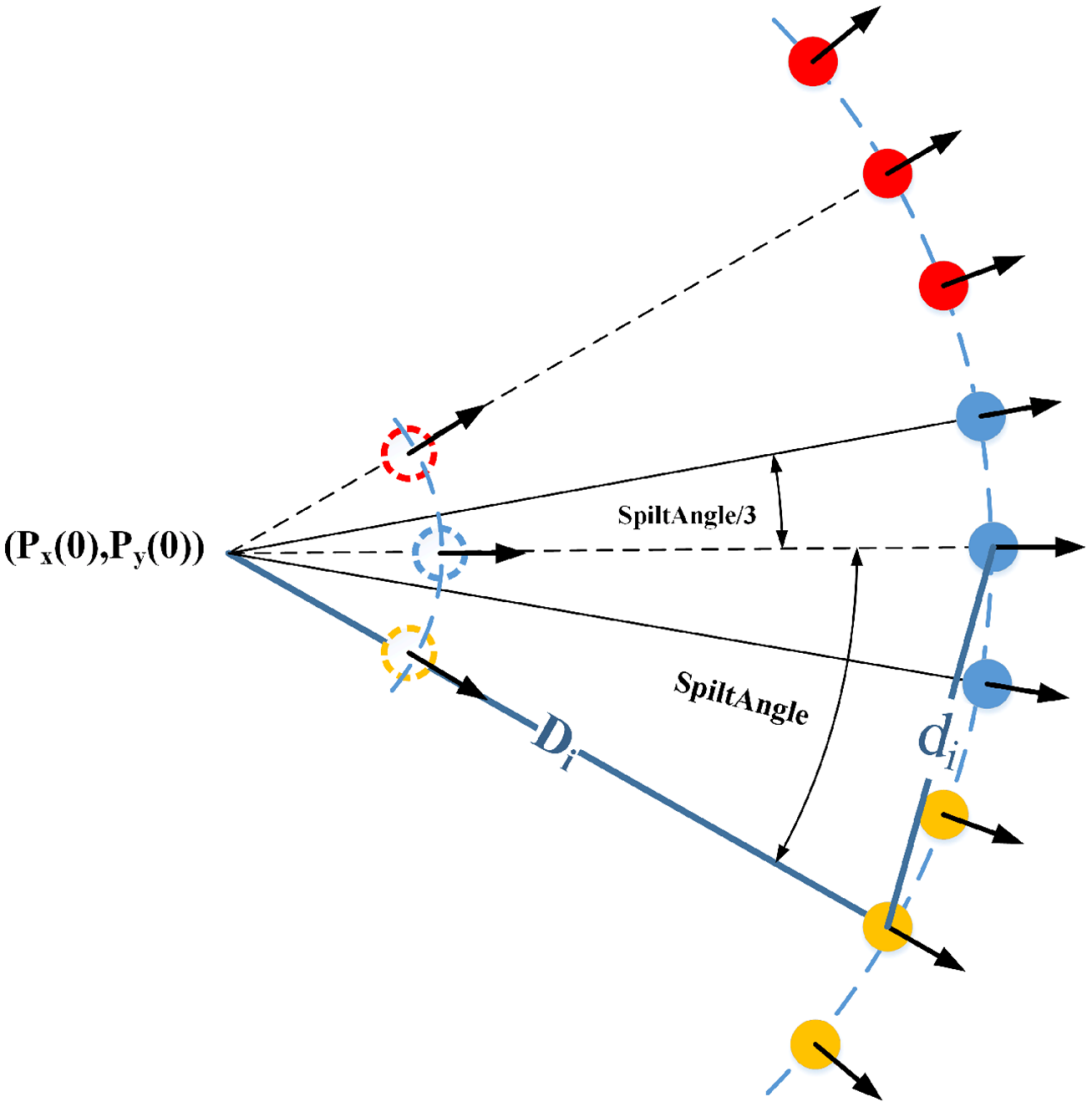
The change before and after particle splitting occurs and the computation of the distance *d*_*i*_ between neighboring particles.

The solution we provided to avoid this phenomenon is to introduce new wave particles in the area between neighboring particles. The new wave particles fill in the gradually widening area between neighboring particles, and thus the overlap between wave particles cannot be destroyed (Fig 5B), an operation process we called splitting of wave particles (Fig 6). A pseudocode description of the different steps in the splitting process is given in Table 1 in Algorithm 1.

**Table 1:** Algorithm 1: Splitting of wave particles.

1:	**for** each wave particle *i* **do**
2:	//compute the distance between the position of particle at time *t* and its position *P*_*x*_(0), *P*_*z*_(0) at time *t*_0_
3:	*D*_*i*_ = |**V**_**i**_ × (*t* − *t*_*born*_) − (*P*_*x*_(0), *P*_*z*_(0))|, **V**_**i**_ = (*cosθ*, *sinθ*);
4:	//compute the distance between neighboring wave particles
5:	*d*_*i*_ = *D*_*i*_ × *SplitAngle*_*i*_;
6:	**while** (*d*_*i*_ > *size*_*i*_/4) **do**
7:	*amp*_*i*_ = /3;
8:	*SplitAngle*_*i*_ = /3;
9:	Duplicate wave particle *i*, introduce new particle *j*, *k*;
10:	**V**_**j**_ = (*cos*(*θ* + *SplitAngle*_*i*_), *sin*(*θ* + *SplitAngle*_*i*_));
11:	**V**_**k**_ = (*cos*(*θ* − *SplitAngle*_*i*_), *sin*(*θ* − *SplitAngle*_*i*_));
12:	**end while**
13:	Add the new wave particles *j*, *k* to Particle List;
14:	**end for**

The distance *d*_*i*_ between neighboring particles determines when we need to introduce new wave particles, and the computation of *d*_*i*_ can be formulated as

$$d_{i}=2\times D_{i}\times \sin\left(SplitAngle_{i}/2\right)$$ (25)

where *D*_*i*_ is the propagation distance of the wave particle. However, when *SplitAngle*_*i*_ is small enough, sin (*SplitAngle*_*i*_/2) = *SplitAngle*_*i*_/2, so Eq 25 can be replaced by the formula in line 5 in Table 1. If the distance *d*_*i*_ between neighboring particles becomes larger than half the particle radius, as shown in line 6, the wave particle *i* will split to produce the new wave particles *j*, *k* on either side. To preserve the overall amplitude of the wavefront unchanged, *a*_*i*_(*t*) the amplitude of particle *i*, should be shared among the three wave particles (see line 7). Similarly, the offset angle between the propagation directions of neighboring particles should reduce to *SplitAngle*_*i*_/3 after the new wave particles are split (line 8).

#### Aperiodicity of wind field

**Fig 7:**
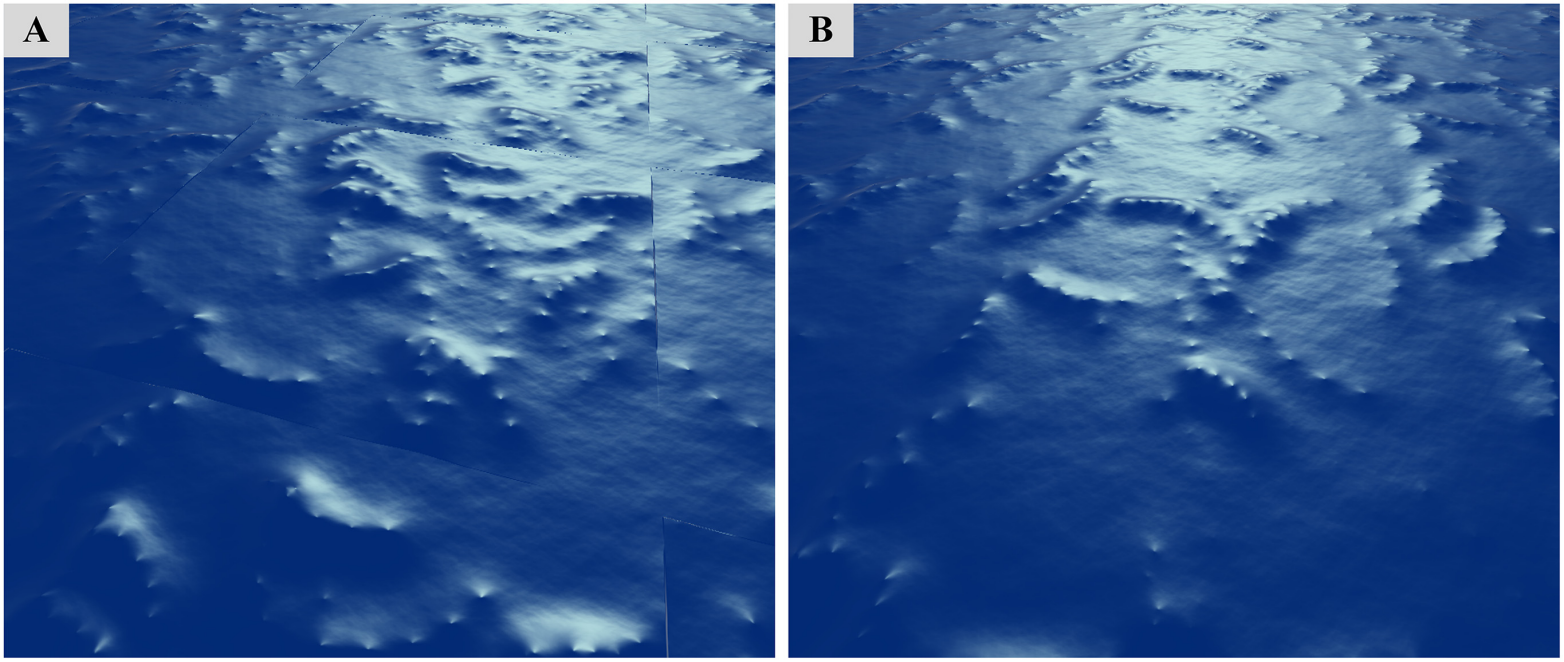
Crack test scenes. (A) Obvious cracks between tiling patches without mirror wave particles. (B) No cracks between tiling patches with mirror wave particles.

The height field of an endless ocean is the result of the tiled extension of patches, which is a popular method for rendering an ocean surface with the limited computational capabilities of current hardware. However, it is an essential premise that the patch must be perfectly periodic, if we need to simulate the endless ocean surface. It should be noted that, unlike the ocean waves produced by the Fast Fourier Transform (FFT) method, the wind field data and the ocean waves driven by such wind fields are in most cases aperiodic. Therefore, the wave particles located on either side of the boundaries of the patch cannot form a complete wavefront, and the resulting crack phenomenon between the patches is obvious. Fig 7A shows the crack problem in an ocean surface tiled by small ocean patches in which the wave particles move randomly.

**Fig 8:**
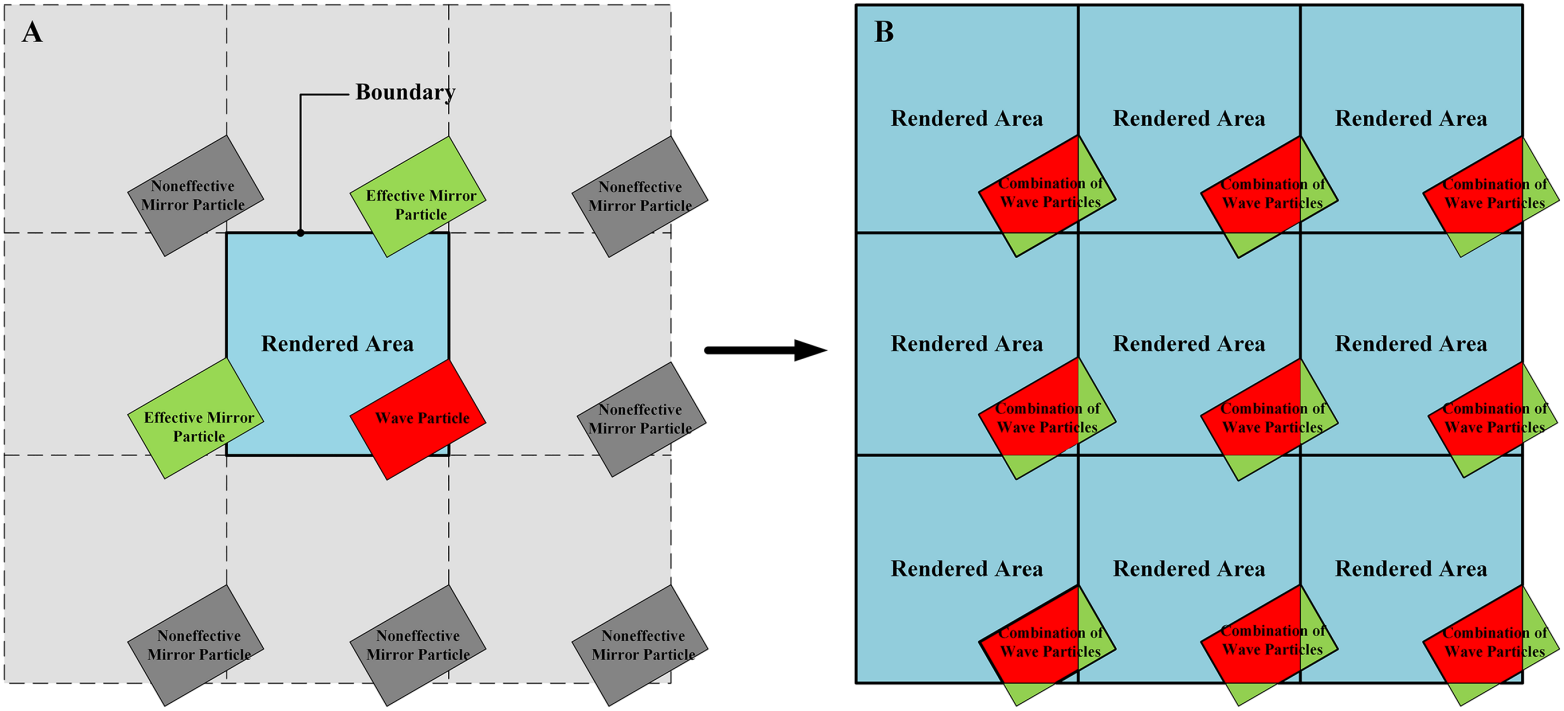
Solution to aperiodicity of wind-driven ocean waves. (A) The relevant location of a reference wave particle and its mirrors. (B) The combination of wave particles located in boundary areas of ocean patches (*Rendered Area*).

The solution we proposed to this problem is to create eight mirror wave particles, which are actually the copies of a reference wave particle (the red box) with the change in position in eight directions, as illustrated in Fig 8. All of these mirror particles and the reference wave particle share the same properties and dynamic behavior, and thus the relevant locations of these particles are fixed. Fig 8A shows that, as the reference wave particle (the red box) crosses the lower and right boundaries of the *Rendered Area*, two effective mirror particles (the green boxes), above and to the left of the reference wave particle, enter the upper and left boundaries. The other six noneffective mirror particles (the gray boxes) that do not enter the *Rendered Area* are neglected to reduce the performance cost. Hence, from the perspective of the whole infinite ocean scene, the wave particles located in the boundaries of the ocean patch consist partly of the reference wave particles themselves and partly of their mirror particles (see Fig 8B). Fig 7B shows that the crack phenomenon is solved by the introduction of the mirror wave particles.

## Results

The following simulations were run on an off-the-shelf PC with an AMD FX-6100 processor (3.3GHz) and a GeForce GTX 550 Ti graphics card. The size of the patch, which can be tiled seamlessly over an endless area, was 1024 × 1024.

**Fig 9:**
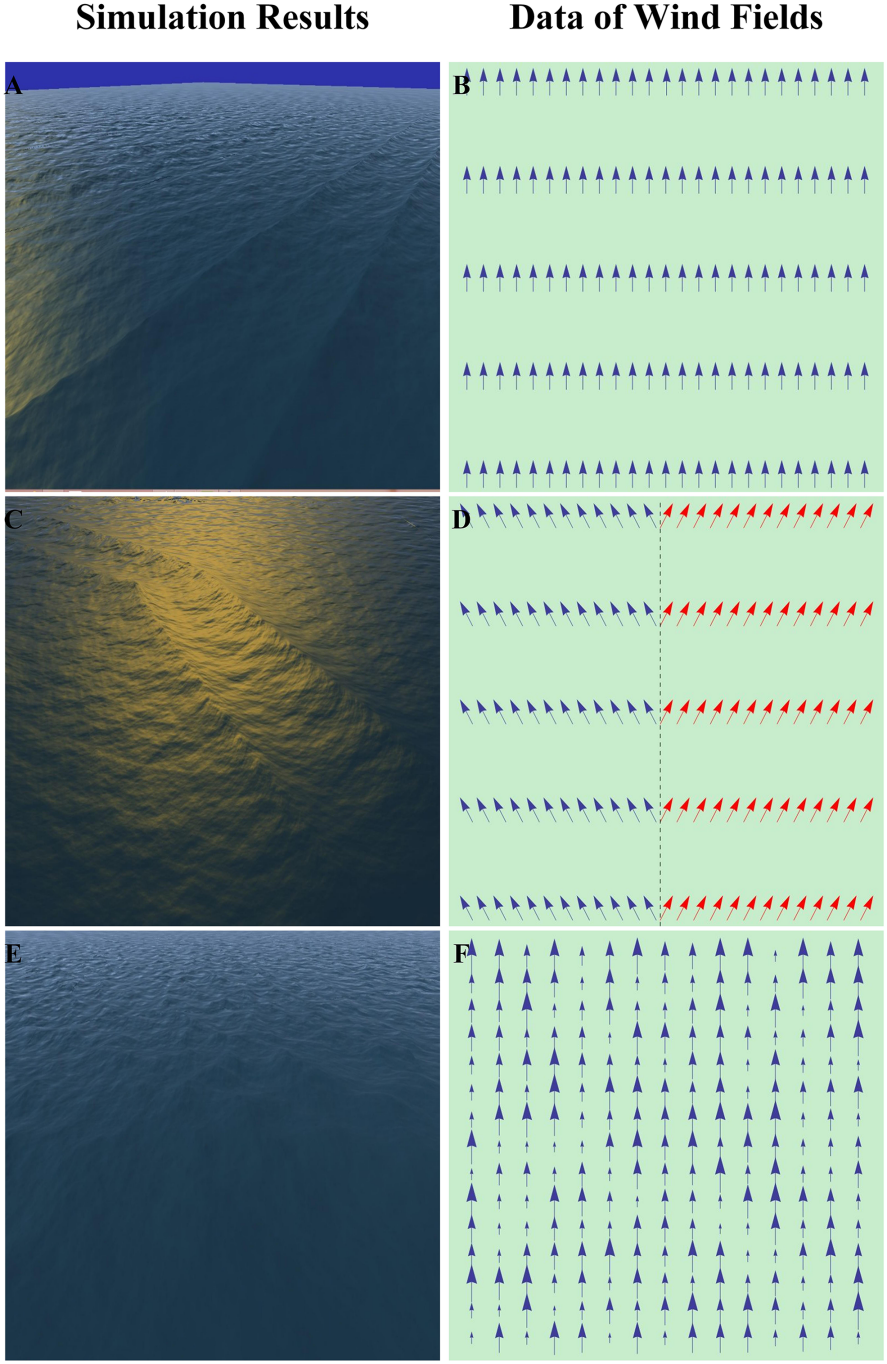
Simulation results under different wind fields. The figures in the left column, (A), (C) and (E), are sample frames captured from the simulation results under the wind conditions of (B), (D) and (F), respectively, in the right column.

To enhance the visualization of the controlling wind field, we simulated several ocean scenes under the conditions of different simple wind fields in Fig 9. The ocean scenes for testing and relevant wind fields are shown in the left and right column, respectively. Fig 9B shows the ocean waves under the wind condition in Fig 9A with identical propagation speed and direction at each sampled point. Fig 9D shows the wind field divided into two areas with the left and right propagation direction but the same speed. Fig 9F provides a wind field with the same propagation direction but different speeds at the sampled points, and Fig 9E shows the controlling effect.

## Conclusion and future work

A novel and efficient method that introduces the concept of wind-driven wave particles into ocean wave construction is proposed in this paper. In most cases of ocean simulation, obtaining a dynamic height map of ocean waves is a complex process, although parallel computing technology is able to speed up the computation. However, via control and management of wave particles driven by wind field data, the height field can be easily and directly converted from the overlap between wave particles without additional computation cost. The final result shows that this approach has the ability to construct various ocean surfaces based on different wind field data.

By constructing various wind fields, we can consider more possible ocean scenes, as the flexibility of the experimental results arises from the flexibility of wind data. For example, in the case of a coastal area near an island, the wind field should be modified to match terrain conditions to provide a possible way to simulate the shallow water. In this way, the ocean waves in both the deep sea and the coastal region could potentially be handled by the same modeling method.

The physical process of the interaction between ocean surface and wind force is complex. We simplified the process so that a reasonable ocean scene could be simulated by our method. However, more experiments and further research on the physical mechanism are needed to improve the realism of the simulation result and to perfect our method.
